# Determine the Role of FSH Receptor Binding Inhibitor in Regulating Ovarian Follicles Development and Expression of FSHR and ER*α* in Mice

**DOI:** 10.1155/2018/5032875

**Published:** 2018-07-09

**Authors:** Luju Lai, Xiaoyun Shen, Haoqin Liang, Yingying Deng, Zhuandi Gong, Suocheng Wei

**Affiliations:** ^1^College of Life Science and Engineering, Northwest Minzu University, Lanzhou, Gansu 730030, China; ^2^State Engineering Technology Institute for Karst Desertification Control, Guizhou Normal University, Guiyang, Guizhou 550001, China; ^3^School of Life Science and Engineering, Southwest University of Science and Technology, Mianyang, Sichuan 621010, China; ^4^Medicine College, Northwest Minzu University, Lanzhou, Gansu 730030, China

## Abstract

Mice of FRBI-1, FRBI-2, and FRBI-3 groups were intramuscularly injected with 20, 30, and 40mg/kg, respectively, for five consecutive days. Ovarian weights of three FRBI groups were reduced in comparison with FSH group. Ovarian cortex thicknesses (OCT) of the FRBI-3 group were less than that of the FSH group (P<0.05). As compared to FSH group, there were fewer numbers of secondary follicles (SFs) and mature follicles (MF) on the ovaries of FRBI-treated mice numbers of primary follicles (PFs) and SFs also decreased. In FRBI-3 mice, we found that the primordial follicles (POF) were scarcer, the follicles developed poorly, and granulosa cells became apoptosis. SF numbers of FRBI-2 and FRBI-3 groups were less than that of the FSH group on day 20 (P<0.05). Maximum longitudinal diameter (MLD) and transverse diameter (MTD) of three FRBI groups became decreased during the experiment. MLD and MTD of the FRBI-3 group were smaller than FSH group. Levels of FSHR mRNA and protein were less than that of CG and FSH group (P<0.05). ER*α* protein levels of FRBI group and serum concentrations of FSH and estradiol (E_2_) in the FRBI-treated mice were decreased when compared to CG and FSH group. In conclusion, FSH treatment could increase the numbers of SF and MF, enhance follicle development, reduce the numbers of SF and MF, and depress the follicular development of mice. Furthermore, FRBI declined the mRNA and protein levels of ER*α* and FSHR in the ovaries and dropped serum concentrations of FSH and E_2_ of mice.

## 1. Introduction

Follicle stimulating hormone (FSH) and estradiol (E_2_) can precisely regulate the female fertility depending on the development of ovarian follicles and final ovulation [1, 2]. The interaction between FSH and its cognate receptor (FSHR) activates multiple signaling pathways leading to steroidogenesis production that modulates the differentiation and proliferation of ovarian granulosa cells [3]. FSHR activates the extracellular signal-regulated kinases (ERK). However, the mechanisms of these actions are unknown [4].

FSH receptor binding inhibitor (FRBI) blocked the combination of FSH into FSHR and inhibited FSH action on at the gene and protein levels [5, 6].* In vivo* administration of FRBI resulted in the suppression of ovulation and induced follicular atresia in mice [7] and impacted the fertility in marmosets [8]. Recently, there has been little information about FRBI effects on follicular development and reproduction functions in human and animals [3, 9]. The exact mechanism of FRBI actions remains still unclear [3, 10].

Estrogen regulates fertility of human and animals. Cellular responses to estrogen are mediated by estrogen receptor *α* (ER*α*) and estrogen receptor *β* (ER*β*) [11].

The binding of estrogen to its receptors (ERs) interacts with nuclear estrogen response elements leading to transcription initiation [12]. In mouse and rat models, disruption of ER*α* causes infertility in both males and females. However, the roles of ER*α* and ER*β* in reproductive function remain undecided [13]. Up to date, it remains unclear if FRBI treatment impacts the expression levels of estrogen receptors in the ovarian follicles [14, 15].

The present work was performed to assess the effects of FSH receptor binding inhibitor (FRBI) on the development of ovaries and follicles and reproduction functions, to understand the FRBI mechanism of inhibiting the interaction of FSH to FSHR in the follicles, and to investigate the signal transduction and pathway of FRBI actions in mice.

## 2. Materials and Methods

### 2.1. Preparation of FSH Receptor Binding Inhibitor (FRBI)

The FSH receptor binding inhibitor (FRBI) peptide of 99.9% purity was synthesized and characterized before being used for the experiments. The preparation of FRBI was performed according to the methods established in our laboratory [4, 8]. The concentration of FRBI was 1000*μ*g/mL.

### 2.2. Animal Treatment

150 Kunming female mice, 21 days old, body weight of 18.00±1.23g, were purchased from Lanzhou University [License No. SCXK (Gansu) 2005-0007]. All mice were randomly allocated to FRBI group, FSH group, and control group (CG) (n=30). FRBI of 20, 30, and 40mg/kg body weight were intramuscularly injected into the mice of FRBI-1, FRBI-2, and FRBI-3, respectively, for five consecutive days. 10IU FSH was intramuscularly injected into mice of FSH group for five consecutive days. 0.2mL saline was injected into mice of CG for five consecutive days. Injections were made in the morning (at 8 to 9 a.m.) each day. Referring to our previous procedure [16], all mice were raised in the group, kept in mice cages, and accurately weighed each day using an electronic balance.

### 2.3. Sample Collections and Measurements

After five mice from each group were injected intramuscularly 0.1mg/kg xylazine on days 0, 7, 10, 15, 20, and 30, respectively, they were killed by cervical dislocation. Bilateral ovaries were aseptically cut using a sharp scalpel. The weight of each ovary was weighed immediately on an electronic balance. The average value was calculated based on bilateral ovaries of each mouse. Meanwhile, blood samples were harvested on days 0, 7, 10, 15, 20, and 30, respectively. Serum was separated and stored at -20°C.

### 2.4. Histological Observations and Measurement of Ovaries and Follicles

Ovaries were fixed in 10% formaldehyde, embedded with paraffin wax, then sliced (5*μ*m), and finally stained with hematoxylin and eosin (H&E). The sections were observed under the light microscope (Leica, Japan). Secondary follicles (3-5 mm in diameter) were found and counted. Microscopic images of the ovaries were photographed. Six sites in each section (5 sections in every group, totaling 150 sites for each group) were measured. The ovarian cortex thickness, maximum transverse diameter (MTD) (MLD), and longitudinal diameter of each secondary follicle were measured, respectively, using Pro Plus 2.0 (MOTIC Company, Hong Kong, China).

### 2.5. Real Time RT-PCR (qRT-PCR) of FSHR and ER*α* mRNAs

The levels of ER*α* and FSHR mRNAs were determined using real time fluorescence quantitative PCR (qRT-PCR) and cloning techniques, so as to evaluate the FRBI effects on expressions of ER*α* and FSHR mRNAs in mouse ovaries.

#### 2.5.1. Primer Design

The primers specific for ER*α* (NM-001302531.1) and FSHR (GenBank accession number: NM-013523.3) were designed with Beacon Designer 7.0 software (Premier Biosoft International, Palo Alto, CA, USA) according to manufacturer's guidelines and Primer-BLAST at NCBI. The reference gene was mouse GAPDH gene (NM-008084.2, HM-043737.1) which was used for normalizing expression levels of target genes [17, 18]. The sequences of the primers used in the qPCR were as follows: FSHR, forward 5′-CGTCCTGATGAGCAAGTTTGG-3′ and reverse, 5′-TGGGCTGATTGACTTAGAGGG-3′; ER*α*, forward 5′-CTTGTGTGTGGACACTCCGT-3′ and reverse, 5′-AAGAAAGGCACAAGGCACGA-3′; GAPDH, forward, 5′-CTTCAACAGCGACACTCACTCT-3′ and reverse, 5′-CCACCACCCTGTTGCTGTA-3′.

The concentrations of the primers (100 nM, 200 nM, 300 nM, and 500 nM) were evaluated, and primer-dimer formation was determined using the melting curve analysis. The primer concentrations only showed dimmer-free reactions were used for the further experiment.

#### 2.5.2. RNA Extraction and cDNA Synthesis

In accordance with the manufacturer's instructions [17, 18], the total RNA was extracted from the ovarian samples using the Trizol reagent (Invitrogen, Beijing, China).

The cDNA was synthesized with the superscript™ first-strand synthesis system for the reverse transcription PCR (RT-PCR) (Invitrogen, Beijing, China).

#### 2.5.3. Fluorescence Quantitative RT-PCR (qPCR)

The expression levels of ER*α* and FSHR mRNAs were determined using qPCR based on our previous methods [4, 17]. The relative level of each mRNA was calculated with the 2^-ΔΔ^Ct method and normalized to GAPDH gene on day 0. The samples were detected in triplicate.

### 2.6. Western Blots of ER*α* and FSHR Proteins in Mouse Ovaries

Western blots were carried out referring to our laboratory methods [19]. The integral optical density (IOD) of the scanned bands was achieved by using Quantity One software (Bio-Rad Company, Hercules, CA, USA). A negative control was performed without primary antibody. The relative contents of ER*α* and FSHR proteins were expressed as the proportion between gray values of ER*α* and FSHR proteins divided by that of *β*-actin.

### 2.7. Detection of Serum FSH and E_2_ Concentrations

Serum concentrations of estradiol (E_2_) and FSH were detected with the especial E_2_ and FSH kits for mice (ELISA) following the operation manual (Cusabio Biotech Co., Ltd., Wuhan, China), respectively. Detection limits were 0.02pg/mL for E_2_ and 0.01ng/mL for FSH. The inter- and intra- experimental variation coefficients were less than 6% and 5%. All samples were tested in triplicate. The detailed methods were reported in our earlier study [17].

### 2.8. Data Statistical Analyses

For each group, all parameters (including OCT, MLD, MTD, levels of ER*β* and FSHR, serum E_2_ and FSH) were calculated on the basis of the data of 5 mice in each subgroup, respectively, using SPSS v. 21.0 (SPSS Inc. Chicago, IL, USA). Data are expressed as means ± SEM. Data from each time point was analyzed separately. All variables of five groups complied with the assumptions for a one-way ANOVA. Supplementary Tukey's post hoc tests were done to determine the pairwise differences after significant differences were identified. The significant level was P<0.05.

## 3. Results

### 3.1. Ovarian Weights and Cortex Thickness of Mice

In order to assess the effects of FRBI administration on ovarian development, the ovarian weight and ovarian cortex thickness (OCT) were detected, respectively. In comparison to the control group (CG). Ovarian weights of FRBI groups were dose-dependently lower than that of FSH-treated group (data omitted). But there was no significant difference between groups.

**Figure 1:**
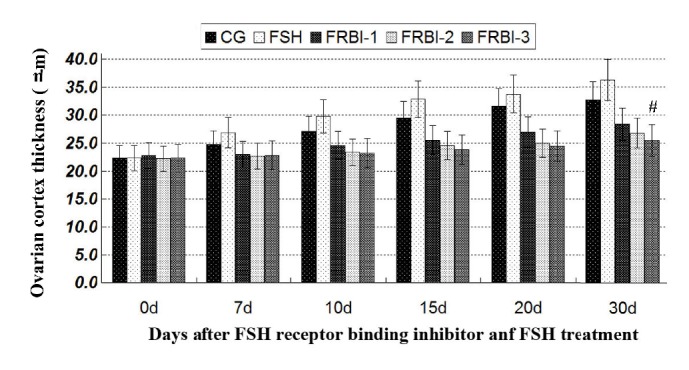
Ovarian cortex thickness of mice. OCTs of three FRBI groups were decreased as compared to CG and FSH group. OCT of the FRBI-3 group was less than that of the FSH group on day 30. ^#^P<0.05 as compared to FSH group.

As shown in Figure 1, OCT of FSH group was slightly increased as compared to CG. OCTs of FRBI groups were reduced in comparison with CG and FSH group, with a minimum value of the FRBI-3 group. On day 30, OCT of FRBI-3 group was less than that of the FSH group (P<0.05). The results demonstrated that a high dose of FBRI (40mg/kg) could reduce OCT of mice.

### 3.2. Histology Structures of Follicles

To determine effects of FRBI regulating FSH function on follicular development, ovarian structure was observed under microscope (×100). Histological changes on day 20 were described in all groups as follows.

#### 3.2.1. Control Group (CG)

**Figure 2:**
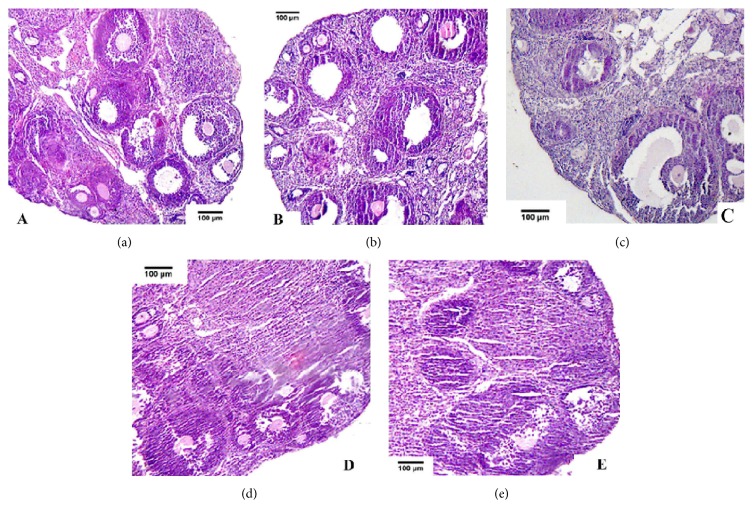
Histological observation of ovaries and follicles on day 20 (×100, scale bar =10*μ*m). (a) Control group (CG); (b) FSH group; (c) FRBI-1 group; (d) FRBI-2 group; and (e) FRBI-3 group.

The primordial follicles (POF) and primary follicles (PF) were small. A few of the secondary follicles (SF) and mature follicles (MF) existed. The structures of the ovaries and follicles were complete. Ovarian cortex and zona pellucida (ZP) were distinct (Figure 2(a)).

#### 3.2.2. FSH Group

The rich SFs and MFs were distributed. POF and PF became larger, and SF numbers were obviously increased in comparison with CG (Figure 2(b)). The follicular antrum was formed in MFs. Dense granulosa cell layers distributed over SF. Follicles developed fully.

#### 3.2.3. FRBI Groups

The ovaries and follicles were structured completely. Fewer numbers of SF and MF existed on the ovaries in comparison with FSH group. The granulosa cells of SFs arranged tightly (Figure 2(c)). In FRBI-2 group, few SF and MF distributed. PF and SF numbers were reduced. Zona pellucida (ZP) became small. Follicles developed poorly. The granulosa cells were found to become apoptosis (Figure 2(d)). POFs were rarer than that of FRBI-3. Scarce SFs and MFs were observed as compared to FSH and CG groups. Follicles developed poorly. The apoptosis of granulosa cells was observed in FRBI-3 group (Figure 2(e)).

These results demonstrated that FSH treatment could increase the numbers of SF and MF, thus enhancing follicle development. FRBI administration reduced the numbers of SF and MF and therefore suppressed the follicle development.

### 3.3. Numbers of Secondary Follicles in Mice

Data in Table 1 showed that numbers of secondary follicles of FSH group were greater than that of CG during the experiment, with the highest increment found on day 20 (P<0.01). However, follicles numbers of FRBI groups were decreased from day 7 when compared to CG and FSH group. On day 20, follicle numbers of three FRBI groups were less than that of the FSH group (P<0.01). In addition, follicle numbers of the FRBI-3 group were lower than CG on days 15, 20, and 30. The findings indicated that FSH promoted follicular development. FRBI administration decreased the numbers of secondary follicles and depressed the follicular development of mice.

### 3.4. MLD and MTD of Secondary Follicles

**Figure 3:**
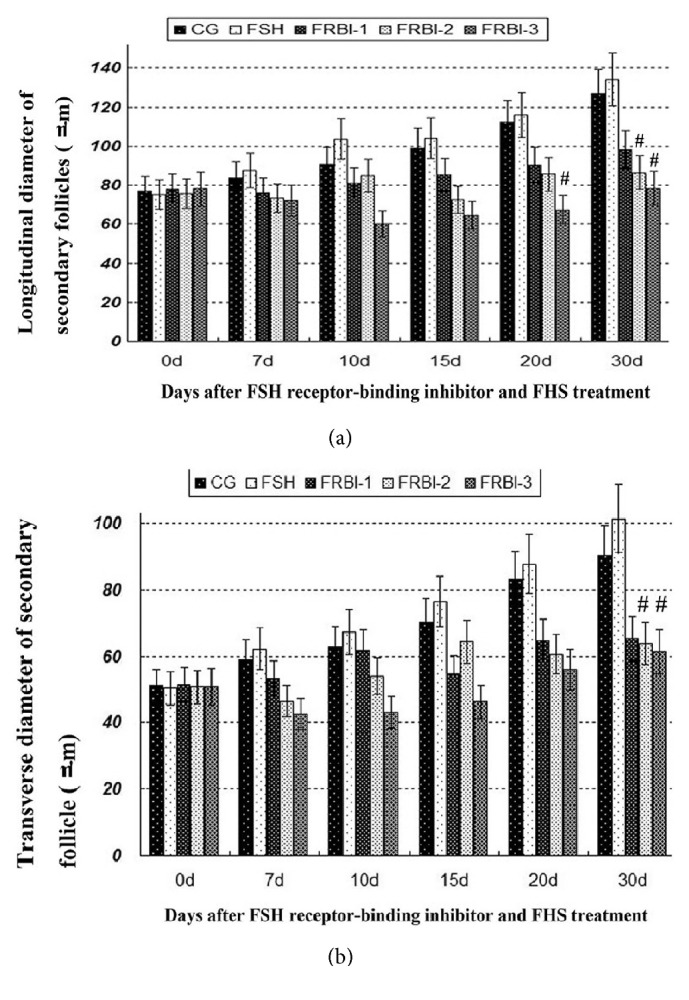
MLD and MTD of mice secondary follicles (×400, *μ*m). (a) Maximum longitudinal diameter (MLD). (b) Maximum transverse diameter (MTD). ^#^P<0.05 as compared to FSH group.

In FSH group, MLD and MTD were increased in comparison to CG (Figure 3). On the contrary, MLD and MTD of three FRBI groups were decreased during the experiment. On day 30, MLD and MTD of FRBI-3 group were significantly lower than those of FSH. The results indicated that FRBI administration could suppress the follicle development of mice.

### 3.5. Expression Levels of ER*α* and FSHR mRNAs in Ovaries on Day 30

**Figure 4:**
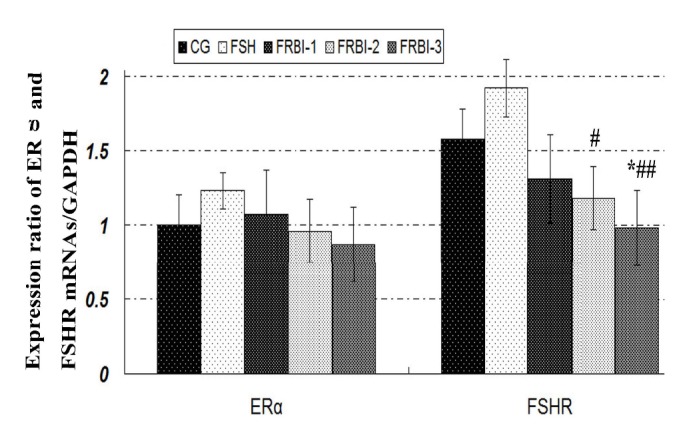
Expression levels of ER*α* and FSHR mRNAs. Levels of FSHR mRNAs were lower than that of CG and FSH group on day 30 after the first FRBI injection. ^**∗**^P<0.05 as compared to CG; ^#^P<0.05 as compared to FSH group. ^##^P<0.05 as compared to FSH group.

As shown in Figure 4, ER*α* and FSHR were expressed in mouse ovaries. Expression levels of ER*α* and FSHR mRNAs were gradually declined in a dose-dependent model. In FRBI-2 and FRBI-3 groups, levels of FSHR mRNAs were lower than CG (P<0.05) and FSH group (P<0.01) on day 30. The findings in our work demonstrated that FRBI treatment reduced FSHR mRNA expression in the ovaries.

### 3.6. Levels of FSHR and ER*α* Proteins in Ovaries

**Figure 5:**
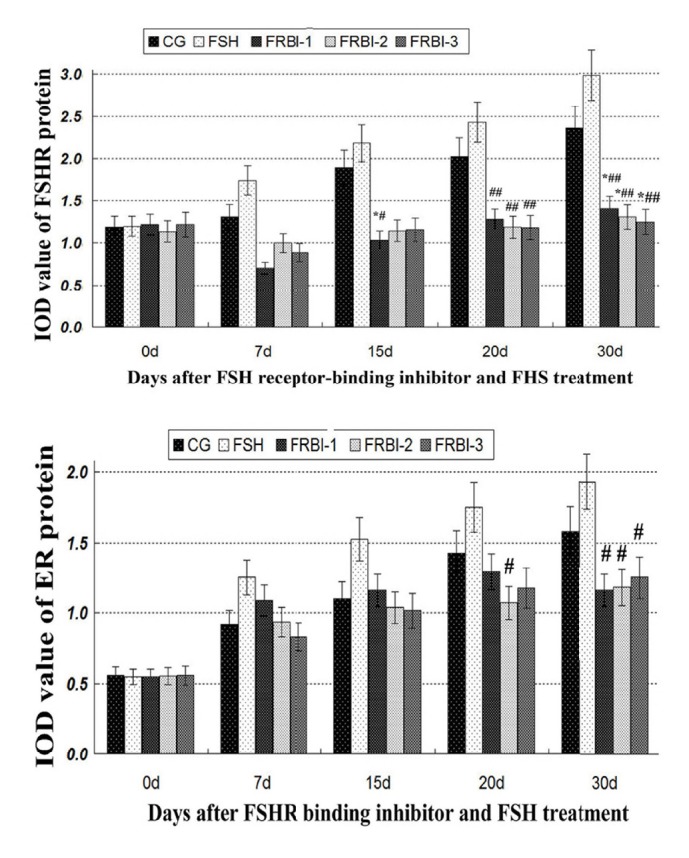
Expression levels of ER*α* and FSHR proteins in the ovaries. ER*α* and FSHR protein levels of FRBI groups were decreased when compared to CG. ^**∗**^P<0.05 as compared to CG; ^#^P<0.05 as compared to FSH group. ^##^P<0.05 as compared to FSH group.

FSHR and ER*α* protein levels were gradually increased in FSH-treated mice from day 7 afterwards of the first FSH treatment (Figure 5). FSHR and ER*α* protein levels of three FRBI groups were reduced in comparison with CG (ER*α* data omitted). FSHR protein levels of FRBI groups were significantly decreased as compared to CG on day 30 (P<0.05) and FSH group on days 20 and 30 (P<0.01). ER*α* protein levels of FRBI group were also less than the FSH group on day 30 (P<0.05). The results exhibited that administration of FRBI suppressed expression of ER*α* and FSHR protein in mouse ovaries.

### 3.7. Serum Concentrations of FSH and Estradiol (E_2_)

Serum FSH concentrations of all mice increased gradually within the experiment. Serum FSH concentration of FSH-treated mice was higher than that of CG (P<0.05) from days 15 (Table 2). FSH levels of three FRBI groups decreased when compared to CG and FSH group. On days 20 and 30, FSH levels of FSH group were higher than that of FRBI-1 (P<0.05) and those of FRBI-2 and FRBI-3 (P<0.01). The findings demonstrated that FRBI could attenuate serum FSH concentrations.

As shown in Table 3, E_2_ concentrations of three FRBI groups were lower than that of FSH group. There was a significant reduction of E_2_ concentrations of FRBI-2 and FRBI-3 on days 20 and 30. The findings revealed that FRBI could decline secretion of FSH and E_2_ of mice.

## 4. Discussion

The development and growth of ovarian follicles are precisely regulated by many genes, such as FSH [2, 20, 21]. FSH acts via cognate FSHR that is mainly expressed by granulosa cells in the follicles [6, 21]. Blocking FSH-FSHR interaction resulted in the decline of FSH action. FSH receptor binding inhibitor (FRBI), as an FSH antagonist, suppressed FSH-FSHR interaction and therefore influenced the efficacy of FSH [5, 22].* In vivo* administration of FRBI resulted in the suppression of ovulation and induced follicular atresia and apoptosis of mice [7, 22] and further impaired the proliferation of granulosa cells [6]. In the present work, the female mice were treated with varying doses of FRBI in order to assess FRBI effects on ovarian and follicular development. FRBI slightly decreased ovary weights, ovarian cortex thickness (OCT), and numbers of ovarian secondary follicles (SF) and mature follicles (MF) on the ovaries. The primordial follicles (POF) and primary follicles (PF) were scarcer. MLD and MTD values of FRBI groups were reduced during the experiment. Follicles developed poorly in the FRBI-treated mice.* In vivo* administration of FRBI depressed the follicle development of mice. Our outcomes were in accordance with previous documents [19, 22]. However, little information has been reported regarding FRBI influences on follicular development in human and animals [3, 10]. The findings in our work still need to be further investigated in the future. The mechanism of FRBI actions also needs thoroughly exploring.

FRBI, a nonsteroidal low molecular weight factor, not only blocked the binding of FSH to FSHR [6], but also altered FSH action of rat granulosa cells at the receptor level [6, 8]. The progesterone (P) secretion was dose-dependently suppressed after the granulosa cells of rats were treated with FRBI in the presence or absence of FSH [6]. FRBI addition into the in vitro maturation medium could decrease the expression levels of FSHR and LHR mRNAs and proteins in cumulus-oocyte complexes (COCs) of sheep [19]. The results in this study indicated that FRBI injection could decline the expression levels of ER*α* and FSHR mRNAs and proteins in the ovaries of mice. The findings were consistent with previous documents [6, 19]. Our findings were in agreement with initial studies [23, 24]. These were probably that FRBI blocked the interaction between FSH and FSHR and, therefore, influenced the efficacy of FSH [5, 6] because FRBI impaired FSH-FSHR combination at the receptor level [22]. The mechanism of FRBI actions has to be thoroughly explored in other animals and humans.

Estradiol (E_2_) is a main circulating estrogen hormone [11]. E_2_ activates the growing large ovarian follicles and promotes follicular growth and differentiation [25]. E_2_ protects granulosa cells from apoptosis and promotes cell cycle progression of healthy follicles [26]. FRBI treatment inhibited progesterone secretion and hampered the growth and differentiation of granulosa cells [8, 22]. Our previous studies indicated that FRBI treatment could suppress FSH production of sheep COCs [19]. The findings in this study showed the serum FSH concentration of FSH-treated mice was higher than that of CG from days 15. But, FSH and E_2_ concentrations of FRBI groups were decreased as compared to CG and FSH group. Therefore, FRBI impacted FSH and E_2_ secretion of mice, which influenced fertility of animals. Our findings were similar to previous document [22, 23, 27]. But they were a disagreement with our initial study in sheep COCs [4]. The actual action of FRBI effects on endocrine function still remains unclear [10, 28] and needs to be investigated.

## 5. Conclusions

FSH treatment could increase the numbers of SF and MF, thus enhancing follicle development. FRBI administration reduced the numbers of SF and MF and depressed the follicular development of mice. A high dose of FBRI (40mg/kg) could reduce OCT of mice. Furthermore, FRBI could drop the mRNA and protein levels of ER*α* and FSHR in the mouse ovaries and decline serum concentrations of FSH and E_2_ of mice. Our study offered a solid basis for thoroughly elucidating the mechanism of FRBI. These will be conducive to promoting ovarian and follicular functions and further to enhance animal's fertility.

## Figures and Tables

**Table 1:** Numbers of secondary follicles (×400).

Group	0d	7d	10d	15d	20d	30d
CG	3.83 ± 0.38	4.50 ± 0.42	4.96 ± 0.43	5.21 ± 0.49	5.65 ± 0.51	6.50 ± 0.59
FSH	3.84 ± 0.33	5.64 ± 0.56	6.27 ± 0.59	7.01 ± 0.63	9.50 ± 0.52^*∗∗*^	7.63 ± 0.62
FRBI-1	3.81 ± 0.35	3.50 ± 0.40	3.23 ± 0.28^#^	4.02 ± 0.39^#^	4.69 ± 0.38^##^	5.06 ± 0.46
FRBI-2	3.82 ± 0.35	3.12 ± 0.32^*∗*#^	3.65 ± 0.38	3.11 ± 0.31^*∗*##^	4.38 ± 0.36^##^	4.55 ± 0.39^#^
FRBI-3	3.81 ± 0.32	2.53 ± 0.37^*∗*##^	3.15 ± 0.27^#^	3.04 ± 0.28^*∗*##^	4.32 ± 0.41^*∗*##^	4.11 ± 0.40^*∗*#^

**Note:**  ^**∗**^P<0.05 as compared to CG; ^**∗∗**^P<0.01 as compared to CG;

^#^P<0.05 as compared to FSH group; and ^##^P<0.05 as compared to FSH group.

**Table 2:** Serum FSH concentrations (ng/mL).

Group	0d	7d	10d	15d	20d	30d
CG	9.74 ± 1.31	12.24 ± 1.14	15.76 ± 1.68	18.67 ± 1.71	22.56 ± 2.23	26.88 ± 2.42
FSH	9.54 ± 1.21	15.44 ± 1.24	21.12 ± 1.88	30.35 ± 3.72^*∗*^	39.88 ± 4.56^*∗*^	45.78 ± 5.73^*∗∗*^
FRBI-1	9.82 ± 1.01	11.68 ± 1.19	19.12 ± 1.79	18.6 ± 1.91	21.27 ± 2.32^#^	30.02 ± 3.42^#^
FRBI-2	9.58 ± 1.09	9.05 ± 1.28	12.24 ± 1.18^#^	13.15 ± 1.13^#^	15.78 ± 1.48^*∗*##^	20.63 ± 2.21^##^
FRBI-3	9.69 ± 1.02	8.08 ± 1.01^#^	7.88 ± 0.77^##^	9.47 ± 1.34^##^	12.28 ± 1.16^*∗*##^	18.64 ± 1.95^*∗*##^

**Note:**  ^**∗**^P<0.05 as compared to CG; ^**∗∗**^P<0.01 as compared to CG;

^#^P<0.05 as compared to FSH group; and ^##^P<0.05 as compared to FSH group.

**Table 3:** Serum estradiol (E_2_) concentrations (pg/mL).

Group	0d	7d	10d	15d	20d	30d
CG	97.6 ± 10.3	107.4 ± 11.5	113.1 ± 12.4	117.4 ± 10.6	120.8 ± 13.8	125.8 ± 12.7
FSH	98.6 ± 9.8	110.6 ± 10.6	119.4 ± 11.3	123.2 ± 12.0	135.6 ± 12.7	146.2 ± 15.2^*∗*^
FRBI-1	96.7 ± 9.6	94.1 ± 8.9	92.4 ± 10.2^#^	98.2 ± 10.8^*∗*#^	102.3 ± 11.1	103.3 ± 11.2^##^
FRBI-2	98.2 ± 10.1	90.2 ± 9.2	80.2 ± 9.1^*∗*#^	93.8 ± 8.5^*∗*#^	99.6 ± 9.6^*∗*##^	117.5 ± 10.3^#^
FRBI-3	98.7 ± 9.9	86.0 ± 7.5	72.6 ± 7.8^*∗*#^	88.6 ± 9.1^*∗*#^	96.4 ± 10.2^*∗*##^	104.6 ± 10.5^##^

**Note:**  ^**∗**^P<0.05 as compared to CG; ^#^P<0.05 as compared to FSH group; and

^##^P<0.05 as compared to FSH group.

## Data Availability

The datasets used and analyzed in the present investigation are available from the corresponding author on reasonable request.
