# Corrigendum to “Determine the Role of FSH Receptor Binding Inhibitor in Regulating Ovarian Follicles Development and Expression of FSHR and ER*α* in Mice”

**DOI:** 10.1155/2020/5464070

**Published:** 2020-09-28

**Authors:** Luju Lai, Xiaoyun Shen, Haoqin Liang, Yingying Deng, Zhuandi Gong, Suocheng Wei

**Affiliations:** ^1^College of Life Science and Engineering, Northwest Minzu University, Lanzhou, Gansu 730030, China; ^2^State Engineering Technology Institute for Karst Desertification Control, Guizhou Normal University, Guiyang, Guizhou 550001, China; ^3^School of Life Science and Engineering, Southwest University of Science and Technology, Mianyang, Sichuan 621010, China; ^4^Medicine College, Northwest Minzu University, Lanzhou, Gansu 730030, China

In the article titled “Determine the Role of FSH Receptor Binding Inhibitor in Regulating Ovarian Follicles Development and Expression of FSHR and ER*α* in Mice” [1], Figure 1 and Table 1 presented results duplicated from the authors' previous article [2]. This was raised to our attention by a reader, and the authors explained that the experiments were designed and performed in two stages.

In the animal experiments of the first stage, three doses (20, 30, and 40 mg/kg) of FSH receptor binding inhibitor (FRBI) were intramuscularly injected into mice, respectively. The authors found FRBI could dose-dependently reduce the ovarian cortex thickness (OCT) and ovarian weights of mice. In the second stage, the authors designed four doses of FRBI in order to verify these outcomes.

The results in Figure 1 and Table 1 represented the results of these experiments, and the main results in each article overlapped except the FRBI-4 group. The duplicated results are accurately reported. The authors apologize that some results were repeated without this being explained, and the editorial board agreed with the publication of this corrigendum.
